# Neuroinflammation in Alzheimer’s Disease (AD) and Glioblastoma (GBM): Shared Mechanisms and Therapeutic Insights

**DOI:** 10.3390/cells15121111

**Published:** 2026-06-19

**Authors:** Karolina Mikołajczak, James Chmiel, Jerzy Leszek

**Affiliations:** 1 Department of Rheumatology and Internal Medicine, Wroclaw Medical University, ul. Borowska 213, 50-556 Wrocław, Poland; 2 Institute of Physical Culture Sciences, Faculty of Physical Culture and Health, University of Szczecin, Al. Piastów 40B Block 6, 71-065 Szczecin, Poland; chmielsoon@interia.eu; 3 Department of Psychiatry, Wroclaw Medical University, 50-367 Wroclaw, Poland; jerzy.leszek@umw.edu.pl

**Keywords:** glioblastoma, Alzheimer’s disease, glioma, neuroinflammation

## Abstract

Introduction: Neuroinflammation is a key feature of both Alzheimer’s disease (AD) and glioblastoma, although it leads to different outcomes in each disorder. In AD, chronic activation of microglia and astrocytes by amyloid-β and tau contributes to neuronal injury and cognitive decline. In glioblastoma, tumor cells exploit inflammatory pathways to create an immunosuppressive microenvironment that supports tumor growth. This review compares the shared and distinct neuroinflammatory mechanisms in AD and glioblastoma and highlights their therapeutic relevance. Materials and Methods: This study was conducted as a narrative review based on a PubMed search performed by three reviewers. English-language articles on AD, glioblastoma, and neuroinflammatory pathways were included, covering original studies, reviews, meta-analyses, and experimental and clinical reports. Keywords included neuroinflammation, microglia, astrocytes, tumor-associated macrophages, inflammasomes, NLRP3, NF-κB, HIF-1α, cytokines, blood–brain barrier, and miRNAs. Due to study heterogeneity, findings were synthesized descriptively. Results: AD and glioblastoma share major neuroinflammatory mechanisms, including microglial and astrocytic activation, cytokine signaling, inflammasome activity, blood–brain barrier dysfunction, hypoxia-related changes, and miRNA regulation. In AD, these pathways promote chronic inflammation, synaptic loss, and neurodegeneration, with NLRP3, NF-κB, and M1-like microglial polarization playing central roles. In glioblastoma, similar pathways are redirected toward tumor progression through tumor-associated macrophages, reactive astrocytes, angiogenesis, immune evasion, and therapy resistance. Key overlapping mediators include IL-1β, TNF-α, NF-κB, HIF-1α, GSK-3β, and selected miRNAs. Conclusions: AD and glioblastoma are connected by common neuroinflammatory pathways, but these processes result in neurodegeneration in AD and tumor support in glioblastoma. Understanding these shared and divergent mechanisms may guide the development of biomarkers and targeted therapies focused on microglia, inflammasomes, cytokines, and immune reprogramming in both diseases.

## 1. Introduction

Neuroinflammatory processes are natural mechanisms essential for the proper functioning of the brain. An appropriate inflammatory response protects the brain from factors such as infections and injuries. This response involves not only glial cells—such as microglia, astrocytes, and oligodendrocytes—but also other immune cells, including macrophages, dendritic cells, and peripheral leukocytes. Particular attention should be paid to the role of microglia, which are responsible for recruiting immune cells, producing pro-inflammatory cytokines, and initiating tissue repair processes, as well as to macrophages, which represent the first line of defense in the central nervous system (CNS). Due to their ability to respond to both pro-inflammatory and anti-inflammatory signals, macrophages contribute to the proper regulation of the inflammatory response and the maintenance of homeostasis [1].

Neuroinflammation has emerged as a critical component of the pathogenesis in both AD and glioblastoma [2,3].

Alzheimer’s disease (AD) is a neurodegenerative disorder of the brain. Its pathogenesis primarily involves two processes: the accumulation of extracellular beta-amyloid (Aβ) deposits and the hyperphosphorylation of intracellular tau protein. Aβ is generated through the cleavage of amyloid precursor protein (APP), the levels of which are increased in the brains of patients with AD. The physiological functions of APP include participation in synapse formation and cellular differentiation. In turn, tau protein is involved in the formation of microtubules, which serve structural and transport functions. In AD, due to phosphorylation processes, tau protein acquires the ability to aggregate, thereby forming neurofibrillary tangles [2].

Glioblastoma is the most common and the most lethal primary tumor of the central nervous system in adults. This tumor is characterized by significant heterogeneity in terms of the cells constituting its microenvironment. In addition to tumor cells, the glioblastoma microenvironment comprises a diverse population of non-neoplastic cells, including inflammatory cells. Such a diverse structure is one of the reasons for the considerable resistance of the tumor to treatment [3].

Both AD and glioblastoma are highly heterogeneous disorders characterized by molecular, cellular, and clinical variability. Consequently, neuroinflammatory mechanisms may differ depending on disease stage, molecular subtype, and microenvironmental context. In this review, we discuss the major inflammatory pathways commonly implicated in these conditions.

Despite their differences, both conditions feature profound activation of the brain’s innate immune cells—microglia and astrocytes—and the release of inflammatory mediators that drive disease progression [2,4]. In AD, misfolded protein aggregates trigger microglial pattern recognition receptors and inflammasome pathways, leading to chronic production of cytokines, e.g., interleukin-1β (IL-1β), tumor necrosis factor-α (TNF-α), interleukin-6 (IL-6), that exacerbate neuronal injury and synaptic loss [5,6]. In glioblastoma, tumor cells actively co-opt and reshape the neuroinflammatory milieu: glioma-recruited microglia and macrophages (together termed tumor-associated macrophages, TAMs) secrete cytokines and growth factors that promote tumor proliferation, invasion, angiogenesis, and immune evasion [4]. Both diseases exhibit breakdown of the blood–brain barrier and elements of hypoxia that further modulate inflammatory responses [7]. This chapter provides a comprehensive overview of the cellular and molecular mechanisms underpinning neuroinflammation in AD and glioblastoma, highlighting shared pathways—such as microglial activation, reactive astrogliosis, cytokine networks, and inflammasome signaling—as well as key divergences in their pathophysiology. Interactions between inflammatory cells and the glioblastoma tumor microenvironment are explored, with a focus on how hypoxic conditions, immune cell phenotypes, and inflammasome activation create a vicious cycle sustaining tumor growth. We also discuss therapeutic implications, proposing that insights from one disease can inform novel treatments for the other. Strategies targeting neuroinflammatory processes (for example, modulators of microglial activity, inhibitors of pro-inflammatory cytokines and inflammasomes, or approaches to reprogram the tumor-supportive immune milieu) hold promise in attenuating AD’s neurodegeneration and in overcoming glioblastoma’s resistance to therapy.

## 2. Materials and Methods

This study was conducted as a narrative review with narrative synthesis of the literature addressing neuroinflammatory mechanisms in AD and glioblastoma, with particular emphasis on shared molecular pathways, disease-specific inflammatory responses, and potential therapeutic implications.

A literature search was performed by three reviewers in the PubMed database to identify relevant publications on neuroinflammation in AD and glioblastoma. The search strategy combined terms related to both diseases and key inflammatory mechanisms, including but not limited to: “Alzheimer’s disease,” “glioblastoma,” “glioblastoma multiforme,” “neuroinflammation,” “microglia,” “astrocytes,” “tumor-associated macrophages,” “inflammasome,” “NLRP3,” “NF-κB,” “HIF-1α,” “blood–brain barrier,” “cytokines,” and “miRNA.” Reference lists of selected articles were also screened to identify additional relevant studies. The PubMed database was last searched on 31 May 2026.

The review included a broad range of publication types, such as original research articles, systematic reviews, meta-analyses, narrative reviews, and relevant experimental and clinical studies, when these contributed to the understanding of neuroinflammatory mechanisms in either disease. No restrictions were placed on publication date, in order to capture both foundational and recent literature relevant to the topic. However, only studies published in English were considered eligible.

Articles were selected based on their relevance to the objectives of the review, namely: (1) the characterization of cellular and molecular mechanisms of neuroinflammation in AD and glioblastoma, (2) identification of overlapping inflammatory pathways and points of divergence between the two conditions, and (3) discussion of therapeutic strategies targeting inflammation-related processes. Given the heterogeneity of the included studies in design, methodology, and outcomes, a quantitative synthesis was not performed. Instead, the findings were summarized and interpreted descriptively through narrative synthesis.

This approach allowed for an integrated comparison of evidence from basic, translational, and clinical research, and was considered appropriate for addressing the broad and mechanistic scope of the present review.

## 3. Cellular and Molecular Mechanisms of Neuroinflammation in AD

### 3.1. Microglial Activation and Innate Immune Triggers

In AD, the accumulation of misfolded proteins acts as a chronic stimulus for the brain’s innate immune system [8,9]. Aβ oligomers and amyloid plaques are recognized by pattern recognition receptors on microglia (such as Toll-like receptors, scavenger receptors, and lectin-like receptors), initiating intracellular signaling cascades that activate nuclear factor kappa-light-chain-enhancer of activated B cells (NF-κB) and other proinflammatory pathways [8,10]. Consequently, microglia in AD upregulate the production of cytokines (e.g., TNF-α, IL-1β, IL-6), chemokines, prostaglandins, and reactive oxygen/nitrogen species, aiming to neutralize and clear the offending agents [8,11,12]. While acute microglial activation may be protective, in AD this activation becomes chronic and dysregulated [13,14]. Over time, microglia assume a primed proinflammatory state (often likened to an “M1”-like phenotype) that is neurotoxic rather than neuroprotective [11,13]. Elevated levels of TNF-α and IL-1β in the AD brain can induce excitotoxicity, synaptic dysfunction, and further neuronal injury [12,15]. These cytokines, via cyclooxygenase and prostaglandin E2 signaling, promote calcium dysregulation and oxidative stress in neurons, contributing to synapse loss and cognitive decline [12,16]. Activated microglia also upregulate complement proteins (e.g., C1q, C3) that opsonize synapses and amyloid deposits; while this aids in debris clearance, it also marks synaptic connections for elimination [14,17], compounding neurodegeneration. Genetic factors modulate these processes: for instance, risk polymorphisms in microglial receptors like triggering receptor expressed on myeloid cells 2 (TREM2) or CD33 impair the ability of microglia to phagocytose Aβ and regulate inflammation, leading to excessive cytokine release and accumulation of toxic aggregates [18,19,20]. Thus, microglial activation in AD, driven by continual exposure to Aβ and tau, transforms from an initially defensive response into a chronic source of neural damage.

A key role in maintaining an active inflammatory state is played by the transcription factor NF-κB [21]. It is activated by pro-inflammatory cytokines such as TNFα and IL-1β, which stimulate microglia to further synthesize these cytokines [22,23]. NF-κB promotes the polarization of microglia toward the M1 phenotype [21].

Recent studies indicate that transforming growth factor beta receptor 3 (TGFBR3) also plays a significant role in the pathogenesis of AD. Both the soluble form of the receptor (sTGFBR3) and total TGFBR3 expression are elevated in the brains of AD patients and in animal models of the disease [24,25]. Suppression of sTGFBR3 expression has been shown to improve cognitive function, enhance transforming growth factor beta (TGF-β) signaling, reduce the pro-inflammatory activation of microglia, and increase their phagocytic capacity [24,25]. In contrast, TGFBR3 overexpression leads to impaired memory and learning abilities, increased β-amyloid accumulation, enhanced neuronal apoptosis, and polarization of microglia toward the pro-inflammatory M1 phenotype [24,25]. Collectively, these findings suggest that increased TGFBR3 activity may contribute to neurodegeneration and neuroinflammation in AD, and that modulation of this pathway may represent a promising therapeutic strategy [24,25].

Another factor involved in the inflammatory response is glycogen synthase kinase-3 (GSK-3), a serine/threonine (S/T) protein kinase, which participates in various cellular processes, including growth and differentiation [26,27]. GSK-3 also phosphorylates NF-κB, thereby modulating its activity [28]. However, its role in the pathophysiology of AD is even more significant—GSK-3 is one of the main enzymes involved in the phosphorylation of tau protein [29]. Moreover, it directly contributes to the formation of Aβ by regulating the cleavage of APP [30]. Elevated levels of Aβ further enhance the activity of GSK-3β, creating a self-perpetuating cycle [31].

### 3.2. Inflammasome Signaling

A pivotal molecular mechanism linking Aβ to microglial cytokine release is the activation of inflammasomes–cytosolic multiprotein complexes that govern IL-1β and interleukin 18 (IL-18) maturation [32]. In AD, the NOD-like receptor family pyrin domain-containing 3 (NLRP3) inflammasome in microglia has garnered particular attention [33]. Fibrillar Aβ is phagocytosed by microglia and induces lysosomal leakage and cathepsin release, providing a trigger for NLRP3 assembly [32]. Concurrently, Aβ engagement of surface receptors (like Toll-like Receptor (TLR2/4)) supplies a priming signal (upregulating NLRP3 and pro-IL-1β expression via NF-κB) [34]. Fully activated NLRP3 inflammasomes cleave pro-caspase-1, which in turn processes pro-IL-1β and pro-IL-18 into their active, secreted forms [32,33]. IL-1β is a potent driver of neuroinflammation and has multiple downstream effects: it reduces synaptic plasticity [35], impairs long-term potentiation [36], and can directly injure neurons [36,37], while also recruiting astrocytes and peripheral immune cells to the site of pathology [38,39]. A striking discovery is that activation of NLRP3 in AD not only produces inflammatory cytokines but also leads to the release of Apoptosis-associated Speck-like protein containing a CARD (ASC) specks (the inflammasome adaptor protein aggregates) [40]. These ASC specks can bind to Aβ and act as “seeds” to promote further amyloid aggregation and plaque growth [40]. In this way, a feed-forward loop is established: Aβ activates microglial inflammasomes, which generate inflammatory mediators and ASC aggregates that accelerate amyloid deposition, thereby sustaining and amplifying microglial activation. Consistent with this model, experimental studies have shown that genetic or pharmacologic inhibition of NLRP3 inflammasomes ameliorates pathology in AD models, reducing IL-1β levels and improving cognitive outcomes [33,41]. The inflammasome pathway thus exemplifies how innate immune mechanisms can directly link to the core neuropathology of AD.

### 3.3. Astrocyte Reactivity and Neuroinflammatory Mediators

Astrocytes, the abundant glial cells that normally support neurons and maintain homeostasis [42], also undergo a transformation in AD. In the healthy brain, astrocytes help regulate neurotransmitter levels, metabolic supply, and blood–brain barrier (BBB) integrity. They contribute to the maintenance of cognitive, sensory, and motor functions [43,44,45]. In AD, however, astrocytes in the vicinity of Aβ plaques and damaged neurons become “reactive,” a state marked by hypertrophy, upregulation of glial fibrillary acidic protein (GFAP), and secretion of inflammatory molecules [46]. Activated microglia often drive this astrocytic response: microglia release factors like IL-1α, TNF-α, and C1q that convert astrocytes to a neurotoxic phenotype termed A1 [47]. These reactive astrocytes lose some of their neurotrophic functions and instead produce proinflammatory cytokines, chemokines, and proteases that can exacerbate neuronal injury [47]. For example, reactive astrocytes secrete IL-1β, TNF-α, and complement components that amplify the inflammatory cascade initiated by microglia [48]. They also contribute to glutamate excitotoxicity by reduced uptake of synaptic glutamate, and the resulting excess glutamate further harms neurons [49]. A major consequence of astrocyte-mediated inflammation in AD is blood–brain barrier impairment [50]. Astrocyte endfeet normally reinforce BBB tight junctions, but inflammatory cytokines (like IL-1 and TNF-α) and reactive oxygen species cause endothelial junction disruption and vascular inflammation [51]. Indeed, reactive microglia and astrocytes in AD release factors that cause the disfunction of the BBB, leading to leakage of blood-derived proteins and even peripheral immune cell infiltration into the brain parenchyma [52]. This vascular compromise not only allows inflammatory cells and molecules to enter the CNS but also reduces cerebral blood flow [52,53]. The resulting chronic cerebral hypoperfusion and hypoxia further aggravate neurodegeneration: hypoxia-inducible factors can increase β-secretase activity [54] (promoting amyloidogenic processing of APP) and upregulate RAGE (receptor for advanced glycation end products) on endothelial cells, which facilitates influx of peripheral Aβ into the brain [55].

### 3.4. Chronic Inflammation and Self-Perpetuating Cycles

A defining feature of AD neuroinflammation is its failure to resolve [33]. Normally, acute inflammation triggers counter-regulatory, pro-resolving mechanisms; however, in AD, the continuous presence of Aβ/tau and possibly external factors (like systemic inflammation or metabolic syndrome) maintains microglia and astrocytes in a chronic inflammatory state [6,56]. Over years or decades, this leads to a self-reinforcing cycle: activated glia cause neuronal injury, releasing additional damage-associated molecular patterns (DAMPS) and neuronal debris that further stimulate microglia [57,58]. In aged individuals (and AD patients typically are elderly), the innate immune system is already primed—aged microglia exhibit exaggerated responses to stimuli and a proinflammatory gene profile [59,60]. Thus, an initial trigger (e.g., mid-life Aβ accumulation or even a systemic inflammatory event) might set off an outsized and lingering neuroinflammatory response in the aging brain [61]. Post-mortem and biomarker studies in AD consistently show upregulated markers of inflammation (cytokines, complement, acute phase proteins) even at early stages of cognitive impairment [62,63,64], indicating that neuroinflammation is not merely a late byproduct but an early and integral part of AD pathogenesis [62]. Therapeutically, this suggests that targeting neuroinflammation—by suppressing harmful cytokines, inhibiting inflammasomes, or enhancing microglial debris clearance—could help break the cycle of damage [33,65]. Indeed, some experimental therapies (e.g., anti-inflammatory drugs, microglial modulators) have shown promise in reducing neurodegenerative changes in preclinical models [33,65,66]. In patients, large trials of nonsteroidal anti-inflammatory drugs (NSAIDs) did not yield clear benefits for established AD, possibly because by the time of clinical symptoms the inflammatory process is entrenched and partly deleterious and partly compensatory [67]. Nonetheless, continued exploration of the neuroimmune mechanisms in AD is uncovering new targets (such as TREM2-agonist antibodies to boost plaque uptake, or NLRP3 inhibitors to reduce IL-1β production) that aim to recalibrate the immune response for therapeutic gain.

### 3.5. The Role of miRNAs

MicroRNAs (miRNAs) are short nucleotide sequences lacking protein-coding function. Various subtypes of miRNAs are present in the human body. Their primary role is the post-transcriptional regulation of gene expression—most notably through binding to mRNA and blocking its translation [68]. Different microRNAs play distinct roles in the pathogenesis of AD [69,70]. miR-125 is associated with neuroinflammatory processes and contributes to the abnormal expression of cell-cycle proteins by reducing the expression of the cell-cycle inhibitor CDKN2A. Furthermore, it inhibits the activation of the p53 protein, which is involved, among other functions, in cellular senescence processes [71,72,73]. Likewise, miR-146a is present at elevated levels in the brains of individuals with Alzheimer’s disease (AD) as a result of reactive oxygen species (ROS) accumulation and NF-κB activation [71,74]. Similarly, the expression of miR-210 is increased in response to oxidative stress [75]. miRNA-139 is involved in microglial activation [76], whereas miRNA-137 regulates the NF-κB signaling pathway [77]. Suppression of miRNA-139 has been shown to reduce AD pathology [76]. In contrast, overexpression of miRNA-137 is associated with inhibition of the NF-κB pathway, thereby attenuating neuroinflammation [77]. It is also worth noting that amyloid-β (Aβ) peptides induce a decrease in miRNA-137 levels [78]. Furthermore, miR-29c-3p, miR-193b, miR-132, miR-103, miR-181c regulate Aβ activity, whereas miR-26b, miR-107, miR-191-5p, miR-23a, miR-9, miR-34a-5p are involved in the regulation of the cell cycle and apoptosis [69,79,80,81]. In addition, miR-26b, miR-483-5p, miR-502-3p modulate tau protein activity, while miR-181c, miR-93, miR-31 influence neuroinflammatory processes [69].

## 4. Cellular and Molecular Mechanisms of Neuroinflammation in Glioblastoma

### 4.1. Glioma-Associated Microglia and Macrophages

In glioblastoma, the innate immune compartment of the brain is hijacked to facilitate tumor growth [4,82,83]. The tumor microenvironment of glioblastoma is heavily infiltrated by immune cells, predominantly microglia and monocyte-derived macrophages, collectively referred to as TAMs [4,82]. These cells can constitute up to 30–50% of the total cells within a glioblastoma tumor mass, making them a significant component of the tumor bulk [84]. Microglia are the resident CNS immune cells present from early development, whereas macrophages are recruited from the bloodstream through a leaky or compromised BBB [4,82]. In glioblastoma, as the tumor disrupts the BBB and releases chemoattractants, blood-derived monocytes readily infiltrate the brain and differentiate into macrophages within the tumor milieu [83]. Although phenotypically similar, resident microglia and infiltrating macrophages may occupy different niches—microglia often localize to the tumor periphery, while peripheral macrophages accumulate more in the hypoxic/necrotic tumor core [82,85]. Both subpopulations, however, are actively engaged by tumor-derived signals and undergo functional polarization that largely benefits the tumor [82,85]. Glioblastoma cells secrete a variety of cytokines and chemokines (such as colony-stimulating factor-1 (CSF-1), monocyte chemoattractant protein 1 (CCL2/MCP-1), chemokine (CC motif) ligand 5 (CCL5), and Granulocyte–Macrophage Colony-Stimulating Factor (GM-CSF)) that not only attract TAMs into the tumor but also induce their differentiation into an immunosuppressive, tumor-supportive phenotype [4,82]. In immunological terms, TAMs in glioblastoma tend to resemble “M2” macrophages: they express anti-inflammatory cytokines (interleukin 10 (IL-10), TGF-β), scavenger receptors, and angiogenic factors, while showing decreased expression of pro-inflammatory M1 markers that would normally help attack tumor cells [4,86]. This polarization is driven by the cytokine environment—signals like interleukin 4 (IL-4), interleukin (IL-13), and interleukin 10 (IL-10) produced within the TME push TAMs toward the M2 state, whereas pro-inflammatory stimuli (interferon γ (IFN-γ), TLR ligands) are either absent or actively suppressed by the tumor [4,87]. As a result, instead of mounting a robust cytotoxic response against the tumor, TAMs contribute to tumor progression [4,82]. They release growth and survival factors (e.g., Epidermal Growth Factor (EGF), Vascular Endothelial Growth Factor (VEGF)), matrix-degrading enzymes (MMPs) that facilitate invasion, and immunosuppressive molecules that dampen T cell responses. For instance, TAM-derived TGF-β and IL-10 inhibit the activation of nearby T cells and natural killer cells, fostering an immune-privileged niche around the tumor [4]. TAMs can also directly stimulate tumor cell motility and proliferation through cytokines like IL-6, IL-1β, and TNF-α in early phases, though chronic exposure often shifts them to a more suppressive mode [4,86]. Notably, TAMs are plastic—they can exhibit a spectrum of activation states [87]. There is evidence that early in tumor development, some microglia/TAMs attempt an M1-like anti-tumor response (releasing TNF-α, interleukin 12 (IL-12), phagocytosing tumor cells), but as the tumor evolves, the balance swings towards M2-like functions due to persistent tumor-derived conditioning [4,82]. The net effect is that TAMs become pivotal enablers of glioblastoma aggressiveness. High infiltration of immunosuppressive TAMs correlates with increased tumor invasiveness and worse patient prognosis, highlighting their role in cancer pathology [4,82].

In glioblastoma, a complex signaling network involving the WNT/β-catenin, GSK3β, and NF-κB pathways plays a central role in regulating tumor cell proliferation, survival, invasiveness, and immune interactions [88,89]. GSK3β serves as a key signaling hub integrating multiple cellular pathways, and its activity is tightly controlled by upstream signaling, including the Phosphoinositide 3-Kinase/Protein Kinase B (PI3K/AKT) axis, which inactivates GSK3β through Ser9 phosphorylation [89,90]. Under physiological conditions, active GSK3β forms part of the β-catenin destruction complex (together with APC and axin), where it phosphorylates β-catenin and promotes its proteasomal degradation, thereby inhibiting T-cell Factor/Lymphoid Enhancer-binding Factor (TCF/LEF)-dependent transcription [89]. In glioblastoma, however, this regulatory balance is disrupted, with frequent AKT activation and consequent GSK3β inactivation, leading to β-catenin stabilization, nuclear translocation, and induction of genes that support proliferation, survival, and malignant progression [89,90].

Glioblastoma is characterized by substantial cellular heterogeneity, including subpopulations of glioma stem cells (GSCs), which exhibit self-renewal capacity, relatively slow proliferation, and high resistance to radio- and chemotherapy. Growing evidence indicates that activation of the WNT/β-catenin pathway represents one of the major mechanisms sustaining stemness, survival, and the tumorigenic potential of GSCs. Consequently, persistent activation of β-catenin signaling promotes the maintenance of the most therapy-resistant cellular subpopulations, which are responsible for glioblastoma progression and recurrence [88].

In parallel, NF-κB is constitutively activated in glioma and contributes not only to tumor cell survival, invasion, mesenchymal transition, and resistance to radio- and chemotherapy, but also plays a significant immunomodulatory role in the tumor microenvironment [88,90]. It promotes microglial recruitment and angiogenesis, facilitates the shift toward a more aggressive mesenchymal phenotype, and regulates immune evasion mechanisms, including modulation of Programmed Death-Ligand 1 (PD-L1) expression, thereby suppressing T cell activity [91]. Interestingly, in tumor-associated macrophages, NF-κB signaling may be downregulated, contributing to their polarization toward an M2-like phenotype that further supports tumor progression [91].

Beyond its role in WNT/β-catenin regulation, GSK3β also exhibits context-dependent and sometimes controversial functions in glioblastoma biology. Depending on the cellular context, it may act either as a promoter or suppressor of oncogenic processes [92]. While increased GSK3β expression has been associated with tumor progression in glioblastoma, its pharmacological inhibition can reduce glioma cell proliferation and survival [92]. Conversely, ectopic expression of GSK3β has been reported to suppress tumor growth, likely through reduced β-catenin signaling [92]. Collectively, these observations highlight GSK3β as a central regulatory node linking WNT/β-catenin and NF-κB signaling, whose dysregulation contributes to both tumor progression and immune modulation in glioblastoma [88,89,90].

### 4.2. Reactive Astrocytes in the Tumor Microenvironment

Astrocytes, which comprise a major fraction of cells in the brain, are not bystanders in glioblastoma pathology [93]. Historically, astrocytes were thought to be passive or even protective in brain tumors, but recent findings indicate that tumor-associated astrocytes become reactive and actively influence glioblastoma progression [94]. In the context of a growing glioblastoma, astrocytes surrounding the tumor undergo astrogliosis—they hypertrophy and upregulate markers like Glial Fibrillary Acidic Protein (GFAP), vimentin, and cytokines, akin to astrocytic changes seen in neuroinflammatory conditions [94,95]. Glioblastoma cells can “activate” astrocytes through secreted factors and direct cell–cell contact, inducing them to support tumor growth [96]. Reactive astrocytes in glioblastoma secrete a host of molecules that benefit the tumor: for example, they release cytokines such as IL-6, IL-8, and chemokines (CCL2, C-X-C Motif Chemokine Ligand 10 (CXCL10), etc.) that can enhance glioma cell proliferation, survival, and migration [96,97]. These cytokines can also reinforce the recruitment and polarization of immune cells (like TAMs) that further assist the tumor [93,98]. Astrocytes are a key component of the BBB and normally help regulate vascular dynamics; in glioblastoma, reactive astrocytes produce vascular endothelial growth factor and other angiogenic signals that contribute to the neovascularization characteristic of high-grade gliomas [96,99]. They also produce matrix metalloproteinases and other proteases that degrade the extracellular matrix, loosening the tissue structure and allowing tumor cells to invade into the brain parenchyma [96,100]. Importantly, astrocyte–tumor crosstalk is bidirectional: glioblastoma cells induce astrocytes to become reactive, and those astrocytes in turn feed back to tumor cells to make them more aggressive [93,95]. One intriguing mode of interaction is through gap junctions and the exchange of ions/metabolites [101,102]. Studies have shown that glioblastoma cells form gap junction connections with astrocytes and hijack ion channels/transporters to modify the microenvironment [103,104]. For instance, glioma–astrocyte interactions via Ca^2+^and K^+^ channels can promote glioma cell invasion by facilitating fluid movement and edema, which create paths of least resistance for cell migration [105,106]. Additionally, astrocytes can buffer or release neurotransmitters (like glutamate) in ways that promote epileptic activity often seen in glioblastoma patients, and glutamate released by tumor or astrocytes can kill surrounding neurons, possibly clearing space for tumor expansion [107].

### 4.3. Other Inflammatory Cells in Glioblastoma

While microglia/macrophages and astrocytes dominate the glioblastoma microenvironment, other immune cells also play roles [108]. The brain is an immune-privileged site, but glioblastoma’s disruption of the BBB and secretion of chemoattractants allow some peripheral immune populations to infiltrate [109,110]. Among lymphocytes, regulatory T cells (Tregs) are notably enriched in glioblastoma tissue despite overall low T cell numbers (~0.5–2% of cells) [111,112]. Tregs, attracted by chemokines like CCL2 and CXCL10, secrete high levels of TGF-β and IL-10, bolstering the immunosuppressive milieu and suppressing cytotoxic T lymphocytes that might otherwise attack tumor cells [113,114,115]. Cytotoxic CD8^+^ T cells and Natural Killer cells (NK cells) are present but often rendered ineffective: glioblastoma cells can express ligands (e.g., PD-L1 or non-classical MHC class I molecules) that inhibit T cell and NK cell activation, and they release TGF-β, which downregulates the lytic activity of NK cells [116,117]. Dendritic cells (DCs), the antigen-presenting cells, are sparse within the tumor and may be functionally impaired by tumor signals, limiting effective initiation of anti-tumor immunity [117,118]. Additionally, a high presence of neutrophils has been observed in glioblastoma, especially in areas of necrosis [117,119]. Neutrophils are drawn by glioblastoma-secreted chemokines like IL-8 (CXCL8) and can enhance tumor angiogenesis and growth by releasing elastases and pro-angiogenic factors [119]. Paradoxically, while neutrophils are part of the acute inflammatory response, in glioblastoma their presence tends to correlate with worse outcomes, possibly because they contribute to a pro-tumoral inflammatory state and tissue damage that the tumor exploits [119,120]. Indeed, an elevated neutrophil-to-lymphocyte ratio in blood is a negative prognostic indicator in glioma patients [119].

### 4.4. Hypoxia, Necrosis, and Immune Modulation

A defining feature of glioblastoma pathology is regions of hypoxia and necrosis within the tumor [121]. As the tumor outgrows its blood supply, pockets of low oxygen form, stabilizing hypoxia-inducible factors (HIFs) in tumor and stromal cells [121,122]. HIF-1α drives the expression of VEGF, leading to the growth of new blood vessels—however, these neovessels are abnormal, leaky, and insufficient, so the tumor remains partially hypoxic [122]. The result is a vicious cycle of hypoxia and necrosis, each exacerbating the other [122]. This has significant immunological consequences [123]. Hypoxic conditions in the glioblastoma microenvironment polarize TAMs further toward an M2 phenotype and induce them to express pro-angiogenic and immunosuppressive genes (HIF-1 can directly upregulate arginase-1 and VEGF in TAMs, for example) [123,124,125]. Furthermore, necrotic tumor cells release DAMPs such as high-mobility group box 1 (HMGB1), ATP, and DNA, which can activate remaining immune cells through receptors like TLRs and inflammasomes [124,126]. One might expect this to trigger anti-tumor inflammation; however, in glioblastoma the necrotic areas are typically surrounded by hyperimmunosuppressive zones—high levels of adenosine (from ATP breakdown) and lactate (from anaerobic metabolism) accumulate, both of which suppress effective immune cell function [124,127]. Necrosis in glioblastoma is also associated with the abundance of immunosuppressive myeloid cells (potentially due to chemoattractants released by dying cells) and the secretion of IL-10 and Prostaglandin E2 (PGE-2), which blunt any pro-inflammatory reaction [128]. Clinically, tumors with extensive necrosis and inflammation tend to be more therapy-resistant and portend a poorer prognosis [129]. In fact, the presence of a necrotic, immunosuppressive core is a histological hallmark that distinguishes glioblastoma from lower-grade gliomas [130].

### 4.5. The Role of miRNAs

Similar to Alzheimer’s disease, miRNAs also play an important role in glioblastoma [131]. One of the best-characterized miRNAs is miR-21, which may function as an oncogene, as its expression has been found to be elevated in glioblastoma [132,133]. Its target gene is SMAD7, through which miR-21 influences tumor invasiveness and resistance to therapy [133].

Conversely, decreased expression of other miRNAs may also promote tumor progression. For example, miR-138, through its target gene CDK6, stimulates cell proliferation; miR-139, via ZEB1, drives migration and invasiveness; and miR-490, through TGIF2, facilitates epithelial–mesenchymal transition (EMT). Similarly, miR-203, acting through FGFR1, promotes mesenchymal transition [133]. Reduced expression of miR-218, through its regulation of STAT3, affects cytokine signaling and consequently neuroinflammatory processes.

miRNAs also regulate the activity of well-known tumor suppressor genes and signaling pathways. For instance, the PTEN gene, which limits cell proliferation by negatively regulating the PI3K/AKT signaling pathway, is inhibited by miR-17-5p, miR-23a-3p, and miR-26a-5p [134,135]. Similarly, the p53 gene is regulated by miR-10p-5p [136]. In turn, miR-143-3p, miR-123-3p, and let-5a-5p regulate the RAS signaling pathway, which contributes to increased cell survival and proliferation by enhancing oncogenic transcriptional activity [136,137]. Another well-known miRNA involved in regulating cellular invasion and migration is miR-let-7 [138].

Additionally, tumor infiltration into surrounding tissues by tumor-associated macrophages (TAMs) is enhanced by factors such as TNF-α, IFN-γ, and Vascular Cell Adhesion Molecule-1 (VCAM-1), with VCAM-1 expression being regulated by miR-181 [91]. A reduced level of miR-181 is associated with increased glioblastoma malignancy [91]. Moreover, an inverse correlation has been observed between miR-340-5p expression and TAM polarization toward the M2 phenotype [91]. Lower levels of miR-340-5p are linked to higher recurrence risk, larger tumor size, and poorer prognosis [91].

Additionally, tumor cells have been found to communicate with the tumor stroma via extracellular vesicles containing miRNAs [139]. For instance, stromal cells release miR-6733-5p, which promotes TAM polarization toward the M2 phenotype [91]. Specific miRNA expression patterns are also characteristic of particular glioblastoma subtypes, highlighting their potential as diagnostic or prognostic biomarkers [139]. Due to their crucial role in tumor development and proliferation, miRNAs may represent important therapeutic targets [133]. Furthermore, both miRNA inhibitors and mimics are being increasingly investigated for their therapeutic potential.

## 5. Shared Pathophysiological Features and Divergences

Both AD and glioblastoma are profoundly influenced by neuroinflammatory processes, yet the context and consequences of that inflammation differ. Below, we delineate key shared features of neuroinflammation in AD and glioblastoma, followed by their important divergences.

### 5.1. Innate Immune Activation

Shared: In both AD and glioblastoma, the innate immune cells of the CNS—microglia and astrocytes—become activated and proliferate, leading to an increased presence of inflammatory mediators in the brain [140,141]. Activated microglia and reactive astrocytes are hallmark findings in AD brains (clustered around plaques and degenerating neurons) and in glioblastoma tissues (infiltrating and surrounding tumor islets) [84,140]. Divergence: In AD, this activation is initially in response to pathogenic protein aggregates and damaged neurons, aiming to clear debris but inadvertently causing bystander damage [8]. In glioblastoma, glial activation is driven by factors released from tumor cells and tissue injury; here, immune cells are often co-opted into tumor-promoting roles [141,142]. Essentially, AD’s microglia are responding to neuron-derived danger signals, whereas glioblastoma’s TAMs are responding to tumor-derived signals.

### 5.2. Chronic Neuroinflammatory Environment

Shared: Both diseases exhibit a chronic, self-sustaining inflammatory environment in the brain [143,144]. Once triggered, neuroinflammation tends to persist: AD involves decades of smoldering inflammation due to continuous deposition of Aβ/tau [145], and glioblastoma, although rapid in clinical course, establishes a persistent inflammatory microenvironment as the tumor continuously perturbs the brain tissue [146]. In both cases, resolution of inflammation is impaired, leading to prolonged glial activation and cytokine production [143,144,147,148]. Divergence: The impact of this chronic inflammation differs. In AD, chronic neuroinflammation contributes to the progressive degeneration of neurons and synapses, correlating with cognitive decline [14]. In glioblastoma, chronic inflammation contributes to tumor maintenance and progression; however, it is a pathological inflammation that favors immune evasion, so the typical destructive potential of inflammation against abnormal cells is subverted [149]. In fact, chronic inflammation in glioblastoma often correlates with worse outcomes not because it kills tumor cells, but because it helps them survive (e.g., by promoting angiogenesis and suppressing T cell responses).

### 5.3. Blood–Brain Barrier Dysfunction

Shared: Both AD and glioblastoma involve significant dysfunction of the BBB, linking peripheral and central inflammation [52,146]. In AD, inflammatory mediators and accumulating Aβ can weaken the BBB [52], leading to leakage of plasma proteins [52] and occasional peripheral immune cell entry [53], as well as reduced clearance of toxins from the brain [150]. In glioblastoma, the BBB is often focally breached by the tumor [146]; new blood vessels are abnormal and leaky [151], and tumor-secreted factors like VEGF actively break down astrocyte–endothelial interactions [152]. This breakdown permits a large influx of bone marrow-derived cells (monocytes, lymphocytes) into the tumor vicinity [144]. Divergence: The degree and purpose of BBB dysfunction differ. AD has a more subtle, diffuse BBB leakiness that exacerbates neuronal injury (e.g., fibrinogen extravasation can trigger microglial activation) [52,153], whereas glioblastoma often has regions of frankly open BBB (hence contrast enhancement on MRI) [154,155] which the tumor exploits to receive nutrients and immune cells that it can manipulate [144,148,156]. Additionally, in glioblastoma the BBB dysfunction is so pronounced that it challenges drug delivery (a clinical issue: some regions have intact BBB shielding tumor cells, others not) [157], whereas in AD, BBB dysfunction might facilitate entry of peripheral inflammation [53,56,158] (e.g., a systemic infection can provoke a stronger CNS inflammatory response in AD due to a leaky barrier) [52,56,159].

### 5.4. Hypoxia and Metabolic Stress

Shared: Hypoxic and metabolic stress conditions are present in both diseases and serve to amplify inflammation. AD brains frequently show cerebral hypoperfusion and microvascular damage [54,160]; chronically reduced oxygen supply can trigger molecular responses similar to inflammation, including increased oxidative stress and upregulation of inflammatory genes [54,160]. Hypoxia in AD can induce β-secretase and γ-secretase, increasing Aβ production [54,160,161], and also stimulate microglia via HIF-1α pathways [162]. In glioblastoma, hypoxia is a hallmark due to rapid tumor growth [142,163]—HIF-1α drives not only angiogenesis but also directly alters the immune microenvironment [142,163] (e.g., promoting TAM recruitment and skewing toward pro-tumor phenotypes) [142,164]. Hypoxic tumor regions become necrotic, releasing DAMPs that incite inflammation [165], and yet the hypoxia-driven factors (like adenosine) concurrently enforce immunosuppression [163,166]. Divergence: In AD, hypoxia is a contributing factor to neuronal injury and likely exacerbates microglial activation [54,162] (hypoxia worsens AD pathology by inducing more Aβ and inflammation) [54,160,161]. In glioblastoma, hypoxia is more a consequence of the tumor that secondarily shapes inflammation [142,163] (hypoxia helps the tumor by selecting for more aggressive, inflammation-tolerant cells and taming immune cells) [142,163,165,166]. The temporal aspect differs too: AD hypoxia builds up slowly from vascular insufficiency, whereas glioblastoma hypoxia can be acute and severe in necrotic cores. Nonetheless, in both conditions hypoxia and inflammation form a vicious cycle—hypoxia triggers inflammation, and inflammation (through vessel damage or inefficient perfusion) can worsen hypoxia.

### 5.5. Microglia Phenotype Plasticity

Shared: Microglia are dynamic and can adopt a spectrum of activation states in both AD and glioblastoma [87,167]. In each disease, there is evidence of both pro-inflammatory (classically activated, M1-like) and anti-inflammatory or alternatively activated (M2-like) phenotypes at work, often in different spatial or temporal contexts [142,167,168,169]. Divergence: In AD, the balance may tilt towards a pro-inflammatory phenotype that is neurotoxic (excess M1-like activity without resolution) [167,169], although some late-stage phenomena include phenotypes aimed at debris clearance and tissue repair [167] (which might be considered “dystrophic” or ineffective rather than truly M2-healing). In glioblastoma, the microglia/macrophages are skewed predominantly towards an M2-like, tumor-supportive phenotype [142,170] due to continuous exposure to tumor-derived IL-4, IL-10, etc. [171,172,173]. Interestingly, should microglia encounter something like a pathogen or certain therapy, they could swing towards M1 and attack tumor cells [169,174]—but glioblastoma actively prevents or short-circuits this [171,172]. In short, AD microglia cause collateral damage in an attempt to clear pathology, whereas glioblastoma microglia often fail to attack the pathology (the tumor) and instead assist it. Both diseases highlight the plasticity of microglia in response to environmental cues, underlining that context (neurodegeneration vs. neoplasm) determines whether microglial activation is detrimental or “misguided.”

### 5.6. Inflammasome and Cytokine Signaling

Shared: The molecular inflammatory pathways show overlap [56,164,173,175]. In both diseases, IL-1β and TNF-α are elevated and contribute to disease processes: IL-1β is neurotoxic in AD and can drive glioma cell invasiveness and TAM polarization in glioblastoma; TNF-α can impair synapses in AD and also support tumor inflammation and angiogenesis in glioblastoma [14,164,176]. NF-κB is a central transcription factor activated in AD (in microglia/astrocytes by Aβ) and in glioblastoma (in TAMs and sometimes intrinsic tumor cells by TNF or other signals), leading to the production of many common mediators [8,87,173]. The NLRP3 inflammasome, as discussed, is a common thread: it is activated in microglia in AD and likely also in glioblastoma’s myeloid cells in response to tumor debris or therapy (radiation can induce NLRP3 activation, leading to treatment resistance) [56,87,173,177]. Divergence: The downstream consequences differ [56,163]. In AD, inflammasome activation directly accelerates neurodegeneration (via IL-1β and ASC specks promoting plaques) [56]. In glioblastoma, inflammasome activation might contribute more to therapy resistance and possibly tumor proliferation rather than initial tumor formation [163,178]. Another divergence is in the role of the adaptive immune system’s cytokines: AD pathology is not heavily driven by lymphocyte cytokines (there’s not much IFN-γ or interleukin 17 (IL-17) involved, as AD is not an autoimmune disease in the classical sense), whereas in glioblastoma, T cell-derived cytokines (though in low amounts) like IFN-γ can appear if there is an anti-tumor response (for example, a brisk IFN-γ response is actually rare in glioblastoma and its absence is notable) [148,159].

### 5.7. Disease Context and Outcome

Shared: Ultimately, both diseases underscore that neuroinflammation can significantly alter disease trajectory [179,180]. Chronic inflammation correlates with disease severity and progression rate in both. Divergence: The end result, however, is fundamentally different: in AD, unchecked inflammation contributes to neuronal loss and cognitive impairment; in glioblastoma, inflammation contributes to malignant progression and failure of immune clearance of the tumor [181,182]. One might say AD is an example of too little resolution of inflammation causing damage, and glioblastoma is an example of too much suppression of inflammation (by the tumor) allowing unchecked growth [183,184]. These outcomes reflect how the brain’s immune system, finely tuned to maintain homeostasis, can be deleterious when faced with chronic protein aggregates or can be manipulated in the face of cancer [185,186].

In summary, AD and glioblastoma share a landscape where innate immune cells and inflammation-related molecules are prominent, but the roles they play are opposite in effect—one drives neurodegeneration, the other supports tumorigenesis [179,187]. Recognizing these commonalities and differences is important because it suggests that therapies need to be context-specific: for instance, inhibiting microglial inflammatory signaling might be beneficial in AD but could be double-edged in glioblastoma (where we might instead want to boost microglial ability to fight tumor) [188,189]. Conversely, strategies to overcome an immunosuppressive environment in glioblastoma (like blocking IL-10 or TGF-β) might worsen neuronal injury if applied indiscriminately in AD (where some anti-inflammatory activity is actually protective to neurons) [190,191]. Therefore, understanding the nuanced interplay of neuroinflammation in each condition allows for more precise and condition-appropriate interventions. Similarities and differences between AD and glioblastoma in the context of neuroinflammation are presented in Table 1.

## 6. Potential Crosstalk Mechanisms: Hypoxia, Microglia, and Inflammasomes

Having dissected the roles of neuroinflammation in AD and glioblastoma separately, it is valuable to consider specific mechanisms that not only are common to both diseases but also might represent points of crosstalk between neurodegeneration and neurooncology. Three such interrelated mechanisms are hypoxia, microglial activation, and inflammasome signaling. These factors form a triad that drives pathology in both AD and glioblastoma, and intriguingly, they influence one another. Here, we explore how each operates in AD and glioblastoma, and how understanding them might illuminate cross-disease insights.

### 6.1. Hypoxia and HIF-1 Signaling

Hypoxia (oxygen deprivation in tissues) is a potent modulator of inflammation and cellular behavior. In AD, chronic cerebral hypoperfusion and microvascular pathology lead to a state of mild, sustained brain hypoxia. This condition has several effects that bridge metabolism and inflammation. HIF-1α becomes stabilized in chronically underperfused brain regions, upregulating genes that can inadvertently exacerbate AD pathology. For example, HIF-1α activation increases the expression of β-secretase (BACE1), the enzyme that produces Aβ from amyloid precursor protein, thereby promoting amyloid generation under low-oxygen conditions [160,161,192]. Hypoxia also reduces the efficiency of Aβ clearance; one mechanism is through induction of RAGE on endothelial cells, as mentioned earlier, which facilitates more Aβ influx from blood and impedes efflux [193,194]. Meanwhile, hypoxia is pro-inflammatory: it can cause microglia to shift into a more inflammatory phenotype (sometimes called “HIF-1α-dependent M1” state), and it causes endothelial cells and neurons to release chemokines that attract microglia and peripheral macrophages [162,195]. Thus, hypoxia in AD amplifies neuroinflammation and amyloid pathology in tandem. In glioblastoma, hypoxia is more severe and localized, but its consequences are analogous in the inflammatory realm. HIF-1α in glioblastoma upregulates VEGF, leading to neovascularization, and also induces expression of immunosuppressive mediators like VEGF, cyclooxygenase-2, and adenosine A2B receptors on immune cells, which dampen their activity [196,197,198]. Hypoxic TAMs often show increased production of VEGF and MMPs, linking inflammation to aggressive tumor behavior [199,200]. Notably, HIF-1α in TAMs promotes a transcriptional program that overlaps with M2 polarization (for instance, inducing arginase-1, which depletes arginine needed for T cell function) [200,201]. Additionally, hypoxia in glioblastoma selects for tumor cells that are more invasive and resistant to apoptosis (a phenomenon known as the “hypoxia-induced phenotype”), which means these cells can better withstand both therapy and immune attack [202,203,204,205]. An intriguing point of crosstalk is that systemic or brain hypoxia could potentially influence both diseases: for instance, obstructive sleep apnea (which causes intermittent brain hypoxia) is a risk factor for cognitive impairment and might create a pro-inflammatory milieu in the brain [206,207,208]; theoretically, chronic systemic hypoxia or ischemia might also facilitate a protumorigenic environment in those predisposed to glioblastoma by inducing pro-angiogenic, pro-inflammatory changes. Clinically, approaches that improve oxygenation (hyperbaric oxygen therapy) have been trialed with limited success in AD [209], and in glioblastoma, breathing high-oxygen gas mixtures has been explored to improve radiotherapy outcomes (since oxygen is a radio-sensitizer) [210].

### 6.2. Microglia as a Central Hub

Microglia are the common denominator in AD and glioblastoma neuroinflammation—they are the sensors and effectors of innate immunity in the brain. In AD, microglia detect Aβ and tau and become chronically activated [8,211], as detailed above; in glioblastoma, microglia (as part of TAMs) detect tumor signals and get co-opted [212,213]. The fascinating aspect is how microglia might behave if faced with both AD pathology and a tumor. Although it is rare for patients to have co-occurring AD and glioblastoma (likely due to differing age ranges and the rapid mortality of glioblastoma), experimentally one might ask: would AD-primed microglia (already activated and pro-inflammatory) be more effective or less effective against a tumor? One hypothesis could be that AD-conditioned microglia, which express high levels of inflammatory cytokines and phagocytic receptors, might initially attack tumor cells more vigorously (since they are less suppressed), potentially slowing tumor growth. However, the tumor’s immunosuppressive signals might still override and convert those microglia. Conversely, if a person had an underlying pro-tumor microglial environment (say, due to some other pathology or immunosuppressive drugs), would that accelerate AD changes? Microglial dysfunction or suppression can indeed worsen amyloid accumulation (since microglia help clear Aβ), implying that a tumor-altered microglial state could impair plaque clearance [214,215,216]. So, there is theoretical crosstalk: microglia cannot simultaneously be optimally neuroprotective and tumoricidal if they are being tugged in opposite directions by AD and tumor stimuli. On a molecular level, microglia in both diseases upregulate certain receptors and signaling pathways. TREM2, a microglial receptor essential for phagocytosis and survival, is upregulated around AD plaques [216,217,218] and also expressed by TAMs in glioblastoma (though its role in glioblastoma is less clear—interestingly, TREM2^+^ macrophages have been noted in some cancers as an immunosuppressive subset) [213,219]. TLRs such as TLR4 and TLR2 mediate Aβ-induced activation in AD [8,211] and also sense DAMPs in glioblastoma’s necrotic areas [220]. The downstream myeloid differentiation primary response 88 (MyD88)/NF-κB signaling is common to both. Thus, drugs targeting microglial activation—for example, small-molecule inhibitors of microglial p38 Mitogen-Activated Protein Kinase (p38 MAPK) or modulators of TLR signaling—could have dual relevance. One crosstalk mechanism of interest is phagocytosis: in AD, microglia attempt to phagocytose Aβ (and later, dead neurons) [214,216]; in glioblastoma, microglia can phagocytose tumor debris and very rarely live tumor cells [168,221,222]. There is a concept called “phagoptosis”, where microglia might be induced to engulf live neurons or tumor cells if certain “eat-me” signals are present [223,224]. In AD, neurons under stress can expose phosphatidylserine or complement tags that microglia recognize, potentially leading to phagocytosis of even viable but stressed neurons—contributing to neurodegeneration [225,226]. In glioblastoma, inducing “eat-me” signals on tumor cells (like exposing calreticulin or using opsonizing antibodies) could prompt microglia/TAMs to phagocytose tumor cells (similar to how macrophages can eat cancer cells when appropriately stimulated) [168,221,222]. This is being attempted in cancer therapy (e.g., CD47-blocking antibodies—CD47 is a “do-not-eat-me” signal often overexpressed on tumor cells). Interestingly, CD47 is upregulated in AD brain on neurons, which might protect them from being cleared too early [227]; if AD therapy targeted CD47 to allow microglia to clear plaques and dead cells, could that approach be translated to glioblastoma to allow TAMs to clear tumor cells? These parallels show microglia are a fulcrum where AD and glioblastoma research can learn from each other. Strategies to modulate microglial activity (either dampening it in AD or enhancing it in glioblastoma in the right way) are a prime example of cross-disease therapeutic concepts.

### 6.3. Inflammasomes—NLRP3 at the Crossroads

The NLRP3 inflammasome has been implicated in both AD and glioblastoma, making it a tangible molecular link. In AD, as described, NLRP3 in microglia contributes to disease progression by releasing IL-1β and facilitating plaque deposition [33,228]. In glioblastoma, evidence suggests NLRP3 can be activated in response to tumor-associated stressors (e.g., surgical injury, irradiation, or chemo) and that it may enable tumor cell survival under those stresses [229,230,231]. Preclinical work suggests NLRP3 signaling contributes to glioma radioresistance, and its inhibition can improve radiotherapy response [231]. Moreover, chronic activation of NLRP3 has been proposed to link brain aging to gliomagenesis, since aged microglia with active inflammasomes create a milieu rich in IL-1β that could be mutagenic or growth-promoting [232,233]. The crosstalk potential here is that NLRP3-driven inflammation might underlie a predisposition to both neurodegeneration and tumorigenesis when occurring in an aged brain. If so, therapies like NLRP3 inhibitors (e.g., the small molecule MCC950, or related caspase-1 inhibitors like VX-765) could serve double-duty: they are being investigated to reduce inflammatory damage in AD and could also be repurposed to diminish pro-tumor inflammation or sensitize glioblastoma to treatments. Additionally, certain inflammasome components like IL-18 might have roles in glioblastoma (IL-18 can promote blood vessel formation and TAM recruitment) [234,235], and IL-18 is elevated in AD and influences amyloid/tau-related and synaptic phenotypes, though its functional role may be context-dependent [236,237]. A fascinating twist is the role of ASC specks mentioned earlier: in AD, ASC specks from microglia promote plaque spread. Could such specks play any role in cancer? Research outside the brain shows that macrophage-derived extracellular ASC/NLRP3 inflammasome particles can be released after pyroptosis and taken up by neighboring cells, where they propagate inflammatory signaling; in some vascular bystander cells, this has been linked to pro-inflammatory and migration-associated phenotypes [238,239,240]. It is not well studied in glioblastoma, but one could speculate that material released by dying TAMs (like ASC aggregates) might affect tumor cells or other stromal cells in ways we do not yet fully understand. From a therapeutic standpoint, if an inflammasome inhibitor enters clinical trials for AD (which is plausible given the interest), one could consider including glioblastoma patients or those at risk of glioblastoma in early safety evaluations, especially if they have conditions of neuroinflammation (e.g., post-treatment tumor inflammation called pseudoprogression might be mitigated by inflammasome inhibitors to reduce edema).

In terms of hypoxia, microglia, and inflammasomes interplay: hypoxia can induce inflammasome activation (HIF-1α can drive IL-1β production, and also hypoxic conditions cause mitochondrial dysfunction that triggers NLRP3) [241,242]. Microglia in hypoxic conditions might thus activate NLRP3 more readily. In glioblastoma’s hypoxic niches, TAM inflammasome activity might be particularly high, which could lead to local IL-1β that paradoxically supports angiogenesis and invasion [229,230]. Meanwhile, in AD, micro-infarcts and hypoxic white matter lesions could be foci where microglia are highly inflammasome-active, potentially seeding new spots of tau pathology (since IL-1β is known to promote tau phosphorylation). All three factors can form a reinforcing loop: hypoxia → microglial activation → inflammasome → IL-1β → further microglial activation and possibly reduced capillary perfusion (IL-1β causes vasoconstriction and endothelial inflammation) → more hypoxia.

### 6.4. Clinical and Research Implications

Understanding these crosstalk mechanisms opens interesting avenues. For example, could enhancing brain oxygenation and blood flow (treating hypoxia) simultaneously alleviate AD progression and make the brain less hospitable to tumor growth? There is evidence that exercise (which increases cerebral perfusion and has anti-inflammatory effects) can modestly improve cognitive function in AD [243] and also reduce cancer risk through immune modulation—exercise increases blood flow and might reduce brain hypoxia and baseline inflammation [244]. Similarly, drugs targeting microglial activation (like Peroxisome Proliferator-Activated Receptor gamma (PPARγ) agonists or colony-stimulating factor-1 receptor inhibitors) have been considered in both domains: a Colony-Stimulating Factor 1 Receptor (CSF-1R) inhibitor (like PLX3397) can deplete TAMs in glioblastoma models and has been tried in patients [245], and interestingly a similar approach of modulating microglia has been tested in AD models [246,247] (though in AD, removing microglia entirely might impair clearance of plaques—so the approach would differ: in AD one might aim to shift microglia to a cleaning but not inflammatory mode, whereas in glioblastoma one might either remove them or drive them to an inflammatory anti-tumor mode). Inflammasome inhibitors, as noted, represent a concrete overlapping therapeutic strategy [234,246]. Moreover, measurement of these factors could serve as biomarkers: inflammation-related analytes, including IL-1β-pathway components, are of biomarker interest in both AD and glioblastoma, but the most mature AD fluid biomarkers currently are other neuroinflammatory markers such as GFAP, sTREM2, and Chitinase-3-like protein 1 (YKL-40) [248,249,250] (in fact, neuroinflammation imaging—e.g., Translocator Protein Positron Emission Tomography (TSPO PET) scans—lights up in both AD and glioblastoma, highlighting areas of activated microglia) [251,252,253].

## 7. Therapeutic Implications and Opportunities for Cross-Disease Strategies

Recognition of the inflammatory underpinnings in both AD and glioblastoma opens up a host of therapeutic opportunities. While the ultimate goals differ—neuroprotective, anti-degenerative therapy in AD versus cytotoxic, anti-tumor therapy in glioblastoma—there is a surprising convergence in the strategies that might be employed to modulate the immune system for benefit. This section discusses how interventions targeting neuroinflammation might be leveraged in each disease, and how a cross-disease perspective can inspire novel approaches.

### 7.1. Anti-Inflammatory and Immune-Modulating Therapies in AD

In AD, an obvious strategy is to quell the harmful inflammation that contributes to neuronal death [67,254]. This has been attempted in various forms [67,254,255,256]. NSAIDs were epidemiologically associated with reduced AD incidence, prompting trials in early AD—however, large trials of NSAIDs (like naproxen or celecoxib) did not show clear cognitive benefits in symptomatic AD [67,254]. It is thought that timing is crucial: anti-inflammatory prevention might need to start years before clinical symptoms [67,254]. Nonetheless, more targeted approaches are being explored [255,257]. One is inhibition of the NLRP3 inflammasome [258]. Preclinical studies using the small-molecule inhibitor MCC950 showed reduced plaque load and improved memory in AD mouse models by blocking IL-1β activation [259]. If drugs like MCC950 advance in development, they could be repurposed for conditions of central inflammation beyond AD—including perhaps glioblastoma, where as noted NLRP3 contributes to therapy resistance [259]. Another approach in AD is the use of cytokine antagonists [257]. Small trials with anti-TNF-α biologics (like etanercept) have been conducted in AD patients, with mixed results and some signals of improved behavior or inflammation markers, but no established efficacy yet [257]. Given TNF is elevated in both AD and glioblastoma microenvironments, neutralizing it could, in theory, protect neurons and simultaneously remove tumor growth stimuli [256]. However, one must be cautious: completely suppressing TNF-α might impair microglial phagocytosis of Aβ or diminish immune defense against the tumor [257,260]. A more refined approach might be using glucocorticoids or other broad immunosuppressants in AD—but steroids like prednisone, while effective at reducing brain inflammation, have cognitive side effects and are generally avoided long-term [255]. Interestingly, dexamethasone is standard care in glioblastoma to reduce edema (mass effect from inflammation), which improves neurological symptoms but at the cost of likely suppressing any anti-tumor immune response; learning from AD, one might surmise that chronic dexamethasone could also worsen cognitive outcomes by affecting synaptic function [261,262]. Thus, better alternatives are needed.

A promising AD-specific strategy is boosting the brain’s innate immune clearance capacity without causing inflammation [258]. This includes microglial activation through TREM2 [263,264]. TREM2 agonist antibodies are under development, aiming to encourage microglia to uptake and degrade amyloid and apoptotic cells more efficiently, ideally shifting them to a protective state [65]. If such an approach works in AD, one could consider whether enhancing microglial phagocytosis might help in glioblastoma—for example, making TAMs better at engulfing tumor cells [265]. As mentioned, blocking the signal CD47 on tumor cells has entered trials for cancers including gliomas; that concept is analogous to enhancing innate clearance, which is what AD therapies like TREM2 agonism strive for (i.e., remove toxic material or cells) [265].

Another cross-cutting idea is resolving inflammation rather than just suppressing it [258,266]. In AD, the chronic inflammation indicates failure of resolution pathways [266]. Therapies could involve pro-resolving mediators (lipoxins, resolvins, protectins) that actively drive the cessation of inflammation and tissue repair [267]. While these have mostly been studied in peripheral inflammation, there’s growing interest in applying them to neuroinflammation [268]. In cancer, interestingly, tumors exploit some resolution signals to tamp down immune attack, but short-term boosting of resolution could also quell tumor-promoting inflammation (for example, statins have anti-inflammatory and microglia-modulating effects, and observational studies suggest statin use may lower glioblastoma risk or improve survival modestly—possibly through anti-inflammatory, anti-angiogenic effects) [269].

Another proposed approach involves the inhibition of NF-κB-related pathways due to their role in directing microglial polarization toward the M1 phenotype [91]. Consequently, agents such as neuregulin-1 (an NF-κB pathway inhibitor acting through the Human Epidermal Growth Factor Receptor 4 (ErbB4) receptor) and sarcodonin A (a compound derived from mushrooms) are being investigated [91]. The role of Dectin-1, a pattern recognition receptor expressed on microglia, is also emphasized—mouse model studies have demonstrated that Dectin-1 knockout is associated with reduced NF-κB pathway activity [91]. This effect is attributed to Dectin-1’s interaction with Aβ, which activates NF-κB and sustains M1 phenotype expression [91].

The potential use of GSK-3β inhibitors in AD has also been considered; however, their efficacy has not yet been confirmed [92].

### 7.2. Reprogramming the Immune Microenvironment in Glioblastoma

In glioblastoma, given the immunosuppressive bent of the inflammation, therapies aim to reprogram or remove the supportive immune elements [270]. One extensively studied method is CSF-1R inhibition (e.g., with pexidartinib or PLX3397), which causes TAM depletion or phenotype shift [271]. Preclinical glioblastoma models responded to CSF-1R blockers with reduced tumor growth and increased survival [272]. However, in early clinical trials, CSF-1R inhibitors did not yield dramatic tumor shrinkage, perhaps due to redundancy of pathways or incomplete reprogramming [271]. There is room to refine this strategy—maybe a combination of CSF-1R blockade with something that stimulates an anti-tumor immune response (like a TLR agonist or a vaccine) could work, essentially “clearing the fog” of TAM suppression and then activating a targeted immune attack [271]. Here’s where AD research is instructive: simply removing microglia (the CSF-1R inhibitors will remove resident microglia too) in an AD model cleared plaques (because no microglia to produce inflammatory factors) but also prevented plaque clearance (because microglia also eat plaques) [273]. In glioblastoma, removing TAMs might leave the tumor unchecked by any immune surveillance at all [274]. So, an alternative is re-educating TAMs [274]. Approaches include Toll-like receptor agonists (like Cytosine–phosphate–Guanine (CpG) oligonucleotides for TLR9, or imiquimod for TLR7/8) delivered into the tumor to stimulate TAMs towards an M1 phenotype [274]. Some early-phase trials have tested intra-tumoral TLR agonists in glioblastoma (often in conjunction with tumor vaccines) and shown that it can increase inflammatory signatures and sometimes produce tumor regression in animal models [275,276]. Another method is blocking the key immunosuppressive cytokines: TGF-β inhibitors (galunisertib, for example) and IL-10/IL-10R blockers are being looked at [277]. TGF-β blockade may release the “brakes” on not just TAMs but also on effector T cells and NK cells [277]. However, TGF-β is also involved in maintaining the extracellular matrix and controlling angiogenesis, so inhibitors can have side effects [277].

Another promising approach could be the suppression of NF-κB due to its role in angiogenesis [91]. Previous studies have demonstrated that the use of a non-degradable mutant Inhibitor of Nuclear Factor Kappa B Alpha (IκBα), which exerts an inhibitory effect on NF-κB, resulted in reduced angiogenesis [91]. However, it is important to consider the complex influence of NF-κB on glioblastoma progression [91].

Given the overlap with AD’s pathways, one exciting cross-disease prospect is NLRP3 inflammasome inhibitors for glioblastoma [278]. As noted, by inhibiting NLRP3 one might reduce radiation-induced tumor recurrence and also potentially mitigate tumor-associated epilepsy and edema (since IL-1β is implicated in seizures and edema formation) [278,279]. Companies developing NLRP3 inhibitors for AD or rheumatoid arthritis could find a niche in glioma therapy, especially during the post-surgery, post-radiation period where a lot of inflammation is present and often deleterious (in AD, you want to reduce inflammation to protect neurons; in glioblastoma, after initial cytoreduction, you might want to reduce inflammation that promotes regrowth while simultaneously boosting immune attack in a targeted way) [270].

Considering the marked heterogeneity of both AD and glioblastoma, the development of personalized immunomodulatory therapies based on inflammatory biomarkers is conceptually attractive but currently remains more feasible as a stratification and monitoring strategy than as a fully established therapeutic paradigm. In AD, inflammatory profiles may vary according to disease stage, amyloid and tau burden, age-related immune priming, vascular comorbidity, apolipoprotein E (APOE)/TREM2-related genetic background, and the balance between protective phagocytosis and harmful chronic cytokine production. In glioblastoma, heterogeneity is even more pronounced because inflammatory signaling differs across molecular subtypes, tumor regions, hypoxic and necrotic niches, blood–brain barrier integrity, treatment phase, and the relative contribution of resident microglia versus infiltrating monocyte-derived macrophages. Therefore, a single inflammatory marker is unlikely to be sufficient for therapeutic decision-making in either disease. A more realistic approach would involve multimodal biomarker panels combining fluid markers, such as IL-1β-pathway mediators, TNF-α, GFAP, sTREM2, YKL-40, cytokine signatures, and extracellular vesicle or miRNA profiles, with imaging-based readouts of microglial activation, including TSPO PET or related neuroinflammatory imaging approaches. In glioblastoma, these markers may also need to be integrated with tumor tissue profiling, radiogenomic features, immune-cell composition, and longitudinal assessment before and after surgery, radiotherapy, chemotherapy, or immunotherapy.

From a clinical perspective, personalized immunomodulation should therefore not be understood as broadly suppressing or broadly activating inflammation, but rather as adjusting the direction, timing, and intensity of immune modulation to the biological context. In AD, the therapeutic goal would generally be to reduce chronic neurotoxic inflammation while preserving or enhancing beneficial microglial functions such as amyloid clearance, debris removal, and tissue repair. In glioblastoma, by contrast, the goal would often be to reverse tumor-supportive immunosuppression, reprogram TAMs toward more anti-tumor phenotypes, and enhance adaptive immune activity without increasing edema, necrosis-associated inflammation, or neurological toxicity. This distinction is critical because the same pathway may have opposite implications depending on disease context; for example, inhibition of IL-1β/NLRP3 signaling may be neuroprotective in AD, whereas in glioblastoma it may need to be combined with strategies that preserve or enhance anti-tumor immunity. Thus, biomarker-guided immunomodulation is feasible in principle, but its clinical translation will require longitudinal validation, disease-specific biomarker thresholds, integration of central and peripheral immune measures, and prospective trials testing whether biomarker-defined inflammatory subgroups respond differently to specific immune-modifying interventions. This approach may ultimately support precision neuroimmunology, but at present it should be framed as an emerging and translationally promising strategy rather than as a clinically mature solution.

### 7.3. Adaptive Immunity and Checkpoint Inhibitors—A Cautionary Note

The success of immunotherapy in other cancers (e.g., melanoma) led to trials of immune checkpoint inhibitors (like anti-PD-1) in glioblastoma [280]. Unfortunately, results so far have been underwhelming in unselected glioblastoma populations, likely because of the low baseline T cell infiltration and the TAM-heavy suppressive environment [280]. Here is an area where AD and glioblastoma diverge strongly—adaptive immunity plays a huge role in cancer but not much in AD. However, one cross idea could be harnessing autoimmunity that arises in one condition to fight another. There have been rare reports of patients developing paraneoplastic neurological disorders (where the immune system attacking a tumor also attacks the brain, or vice versa) [281]. If we understood how to safely induce an immune response against brain-specific antigens in glioblastoma (without causing encephalitis), or conversely, if having AD’s immune milieu (with high innate activation) could present more antigens to the adaptive immune system (perhaps fragments of neurons, etc.), could it trigger a response that might incidentally attack a nascent tumor? It is speculative, but one could imagine, for instance, a vaccine strategy in glioblastoma that involves not just tumor antigens but also adjuvants that mimic an Alzheimer-like inflammation to draw in innate cells [282].

### 7.4. Translational Therapeutic Approaches Based on Shared Neuroinflammatory Pathways

From a translational perspective, the shared neuroinflammatory architecture of AD and glioblastoma suggests several therapeutic opportunities that may be relevant across neurodegenerative and neuro-oncological contexts. Common pathways such as microglial and macrophage activation, NLRP3 inflammasome signaling, NF-κB-dependent cytokine production, HIF-1α-related hypoxic responses, BBB dysfunction, TREM2-related phagocytic signaling, CSF-1R-dependent myeloid-cell survival, and CD47-mediated “do-not-eat-me” signaling represent potential therapeutic nodes that could be exploited for drug development. In AD, these pathways could be targeted to reduce chronic neurotoxic inflammation, limit IL-1β/TNF-α-mediated synaptic injury, enhance controlled clearance of Aβ and cellular debris, and preserve neuronal function. In glioblastoma, similar pathways may be targeted with a different therapeutic intention: to reprogram tumor-associated microglia/macrophages, reduce tumor-supportive inflammation, overcome immunosuppression, improve responses to radiotherapy or chemotherapy, and facilitate anti-tumor immune activity. Thus, cross-disease therapeutic translation does not imply that the same intervention should be applied identically in both disorders, but rather that shared inflammatory mechanisms can guide the identification of adaptable therapeutic targets.

Several examples illustrate this principle. NLRP3 inflammasome inhibition may be neuroprotective in AD by reducing IL-1β release, ASC speck propagation, and inflammatory amplification, whereas in glioblastoma it may be explored as an adjunctive approach to reduce therapy-induced inflammatory resistance and tumor-promoting myeloid signaling. Similarly, modulation of TREM2 or other phagocytic receptors could support amyloid and debris clearance in AD, while in glioblastoma related phagocytic pathways may be redirected toward tumor cell recognition and removal. CSF-1R-targeting strategies, which influence microglial and macrophage survival and phenotype, may also have dual relevance, although their optimal use would differ between diseases: in AD, excessive depletion or suppression of microglia may impair protective clearance functions, whereas in glioblastoma, reducing or reprogramming tumor-supportive TAM populations may enhance anti-tumor efficacy. Likewise, CD47 blockade and related pro-phagocytic strategies may be particularly attractive in glioblastoma, where tumor cells exploit anti-phagocytic signals, but such approaches would require much greater caution in AD because excessive phagocytic activation could theoretically worsen synaptic or neuronal loss.

The major translational challenge is therefore not simply to identify shared inflammatory targets, but to define disease-specific immune states in which modulation of these targets is beneficial rather than harmful. AD and glioblastoma differ profoundly in tempo, cellular context, therapeutic goals, and acceptable risk. In AD, long-term safety, preservation of cognition, avoidance of excessive immune suppression, and maintenance of beneficial microglial repair functions are central concerns. In glioblastoma, the clinical priority is often to overcome an immunosuppressive tumor microenvironment and improve survival, but this must be balanced against the risks of edema, neurotoxicity, steroid use, treatment-related inflammation, and BBB-related delivery limitations. Future translational strategies should therefore combine mechanistic target selection with biomarker-guided patient stratification, longitudinal monitoring of inflammatory activity, and careful timing relative to disease stage and treatment phase. In this sense, shared neuroinflammatory pathways provide a valuable framework for therapeutic development, but successful clinical translation will require precision immune modulation rather than broad anti-inflammatory or pro-inflammatory intervention.

## 8. Summary

Both AD and glioblastoma exemplify brain disorders in which neuroinflammatory mechanisms play a crucial role—contributing to neurodegeneration in AD and supporting tumor progression in glioblastoma. Microglia, inflammasomes, hypoxia, and blood–brain barrier dysfunction are common pathophysiological elements that, however, operate in opposing directions within each condition. Differences in the regulation and course of inflammation highlight the critical importance of the microenvironment and the context of immune system activation. Comparative analysis reveals that identical pathways (e.g., NLRP3, HIF-1α, TREM2) may be associated with vastly different outcomes depending on the disease, underscoring the necessity for immunomodulatory therapies to be precisely tailored to the disease context.

One promising avenue for further development is the identification of biomarkers—such as IL-1β levels or microglial activity assessed via PET imaging—that could enable monitoring of inflammatory status and adjustment of treatment accordingly. The development of drugs targeting inflammasomes, immunometabolism, and signaling pathways involving TREM2, CD47, or CSF-1R holds potential benefits for both patient groups, provided that the distinct immunological needs of each disease are taken into account. Research into the commonalities between neurodegeneration and oncogenesis may open new therapeutic pathways, for example, improving cognitive symptom management in patients with brain tumors.

Understanding the neuroinflammatory interplay between glioblastoma and AD offers the opportunity to develop novel, more effective, and safer therapies for both diseases. Only through a holistic perspective on neuroinflammatory mechanisms can we better comprehend when and how the brain’s immune system becomes an adversary and when it may act as an ally.

## Figures and Tables

**Table 1:** Similarities and Differences between AD and glioblastoma in the context of neuroinflammation.

Feature	AD	Glioblastoma
Triggering factor	Aβ plaques and tau pathology [8]	Presence of malignant tumor cells [141,142]
Inflammatory environment	Chronic neuroinflammation associated with neurodegeneration [143]	Tumor-associated inflammation promoting immune evasion and tumor growth [143,144]
Outcome of inflammation	Cognitive decline and neuronal loss [14]	Tumor progression and therapy resistance [149]
BBB dysfunction	Moderate BBB disruption caused mainly by inflammation [50,52]	Severe but regionally heterogeneous BBB disruption associated with tumor invasion [83,153,154,155]
Hypoxia and metabolic stress	Secondary to inflammation and BBB dysfunction [50,54]	Mainly caused by rapid tumor growth and mass effect [83,160]
Disease course	Slowly progressive chronic disorder [145]	Rapidly progressive and aggressive malignancy [146]
Microglial/macrophage phenotype	Predominance of pro-inflammatory M1 phenotype [167,169]	Predominance of immunosuppressive M2 phenotype [142,170]
Pro-inflammatory cytokines	↑ IL-1β, TNF-α and other inflammatory mediators [14,176]	↑ IL-1β, TNF-α and tumor-promoting cytokines [164,176]
Inflammasome activation	Promotes IL-1β release, ASC speck formation, and neurodegeneration [56,173]	Supports tumor proliferation and progression [163,177,178]
Role of lymphocyte cytokines	Limited contribution to pathogenesis	Minor but significant immunomodulatory role [148,159]
Inflammation balance	Insufficient control of inflammation [183]	Excessive immunosuppression within the tumor microenvironment [184]
Role of miRNAs	Different miRNAs are implicated in neurodegeneration by regulating Aβ activity, participating in the regulation of the cell cycle and apoptosis, modulating tau protein activity, and influencing neuroinflammatory processes [69,76,77,79,80,81]	Different miRNAs influence tumor cell invasiveness, proliferation, and migration, as well as the response to therapy [91,133]

## Data Availability

No new data were created or analyzed in this study. Data sharing is not applicable to this article.
